# Characterization of complex photosynthetic pigment profiles in European deciduous tree leaves by sequential extraction and reversed-phase high-performance liquid chromatography

**DOI:** 10.3389/fpls.2022.957606

**Published:** 2022-10-12

**Authors:** Fanny Petibon, Guido L. B. Wiesenberg

**Affiliations:** Department of Geography, University of Zurich, Zurich, Switzerland

**Keywords:** leaf pigments, chlorophyll derivatives, carotenoid derivatives, sequential extraction, untargeted liquid chromatography-optical detection

## Abstract

Leaf pigments, including chlorophylls and carotenoids, are important biochemical indicators of plant photosynthesis and photoprotection. In this study, we developed, optimized, and validated a sequential extraction and liquid chromatography-diode array detection method allowing for the simultaneous quantification of the main photosynthetic pigments, including chlorophyll a, chlorophyll b, β-carotene, lutein, neoxanthin, and the xanthophyll cycle (VAZ), as well as the characterization of plant pigment derivatives. Chromatographic separation was accomplished with the newest generation of core–shell columns revealing numerous pigment derivatives. The sequential extraction allowed for a better recovery of the main pigments (+25 % chlorophyll a, +30 % chlorophyll b, +42 % β-carotene, and 61% xanthophylls), and the characterization of ca. 5.3 times more pigment derivatives (i.e., up to 62 chlorophyll and carotenoid derivatives including isomers) than with a single-step extraction. A broad working range of concentrations (300–2,000 ng.mL^−1^) was achieved for most pigments and their derivatives and the limit of detection was as low as a few nanograms per milliliter. The method also showed adequate trueness (RSD < 1%) and intermediate precision (RSD < 5%). The method was developed and validated with spinach leaves and their extracts. The method was successfully performed on leaf pigment extracts of European deciduous tree species. Within a case study using *Fagus sylvatica L*. leaves, pigment derivatives revealed a high within-individual tree variability throughout the growing season that could not be detected using the main photosynthetic pigments alone, eventually showing that the method allowed for the monitoring of pigment dynamics at unprecedented detail.

## Introduction

Chlorophylls and carotenoids are the most widespread and abundant plant leaf pigment families and fulfill vital physiological functions, including photosynthesis and photoprotection. Changes in leaf chlorophyll and carotenoid composition have been observed within trees across phenological stages (Polle et al., 2001; Kraj and Zarek, 2021) and a light canopy gradient (Scartazza et al., 2016), as well as in response to a variety of biotic (Fleischmann et al., 2004; Dhami and Cazzonelli, 2020) and abiotic stress (Esteban et al., 2015), including heatwave and drought (García-Plazaola and Becerril, 2000a; Gallé and Feller, 2007; García-Plazaola et al., 2008), UV-B (Šprtová et al., 2003; Láposi et al., 2005, 2009), elevated CO_2_ and O_3_ (Lütz et al., 2001; Haberer et al., 2007; Herbinger et al., 2007), revealing the performance and acclimation capacity of the plant photosynthetic apparatus.

These pigment responses to environmental impacts are typically investigated by measuring the optical properties of leaf chlorophylls and carotenoids that are related to their chemical structure. Chlorophylls (*chls*) are photosynthetic tetrapyroles, whose absorption spectra reflect the presence or absence of a central metal and the nature of substituents (Taniguchi and Lindsey, 2021). Chlorophyll a (*chl a*), which is the most abundant form of chlorophyll in terrestrial plants, presents two absorption maxima at 430 nm and 664 nm in acetone–water (90:10, v/v) (Lichtenthaler, 1987). Chlorophyll b (*chl b*), which differs from *chl a* by one aldehyde group substituent, presents two absorption maxima at 460 nm and 647 nm in acetone–water (90:10, v/v) (Supplementary Figures 1A,B) (Lichtenthaler, 1987). All compounds with similar absorption maxima, i.e., with an altered structure of *chl a* or *chl b* (including isomers of these structures) that can be clearly separated by chromatographic methods are hereafter referred to as chlorophyll derivatives (*chl dev*). Carotenoids (*car*) are characterized by absorption maxima ranging from 400 to 550 nm (Lichtenthaler, 1987). *Car* is categorized into two compound classes: carotenes, and xanthophylls (Dhami and Cazzonelli, 2020). Carotenes are tetraterpenes counting two main compounds, α-carotene (α*-car*) and β-carotene (β*-car*), as well as their precursors, cis-carotenes and all-trans-lycopene (Supplementary Figures 1C,D). Xanthophylls (*xan*) are oxygenated tetraterpene derivatives. Five xanthophylls are found in all green plants with few exceptions: lutein (*lut*), neoxanthin (*neo*), violaxanthin (*vio*), antheraxanthin (*ant*), and zeaxanthin (*zea*) (Supplementary Figures 1E–H). *lut* and *neo* act as anion scavengers, while *vio, ant*, and *zea* form the xanthophyll cycle (VAZ). A large diversity of naturally occurring carotenoids (more than a thousand) supporting photosynthesis, photoprotection, and signaling functions has furthermore been inventoried (Britton et al., 2004; Yabuzaki, 2017).

Leaf chlorophylls and carotenoids have been characterized through a variety of methods taking advantage of their optical properties. This includes (i) *in situ* optical measurements of leaves using a plant probe coupled with a field spectroradiometer (Sims and Gamon, 2002), (ii) light absorption by bulk pigment extracts using a spectrophotometer (Lichtenthaler, 1987), and (iii) light absorption of individual pigments separated with liquid chromatography coupled with an optical detector. In ecophysiological studies focusing on forest trees, the light absorption of the bulk pigment has been preferred to quantify the concentration of *chl a, chl b*, and *car* (Supplementary Table 1) and calibrate optical measurements performed with a field spectroradiometer or airborne spectral sensors (Croft and Chen, 2018). The dynamic of pigment derivatives has consequently been neglected. Yet, Esteban et al. (2015) showed in a meta-analysis that *chl a*+*b* concentrations are constrained between 1.2 and 15.6 μmol.g^−1^d.w. (in ca. 800 green plant species) and that pigment ratios (e.g., *chl a:chl b*, β*-car:chls*, and *neo:chls*) show little to no responsiveness to varying environmental conditions. Insight into the functionality of the photosynthetic apparatus is thus limited by the concentration range and responsiveness of targeted chlorophylls (i.e., *chl a* and *chl b*) and carotenoids. A more comprehensive characterization of the pigment composition (i.e., including pigment derivatives) using chromatographic techniques would offer new insights into plant physiology and a better understanding and calibration of leaf optical properties. However, to the best of our knowledge, no such method is available for forest tree species.

Approaches using liquid chromatography were first developed in the early 1980s (Schwartz et al., 1981). The development of C18 and C30 chromatographic columns has enabled the identification of an increasing number of carotenoids (Gupta et al., 2015) as well as the separation of structurally similar carotenoid isomers [e.g., *lut* and *zea* (Craft, 1992), α*-car* and β*-car* (Nyambaka and Ryley, 1996), astaxanthin and phytoene isomers (Jin et al., 2017)]. Simultaneous characterization of chlorophylls and carotenoids was successively applied to higher plant tissues (García-Plazaola and Becerril, 1999). Most of those methods however target a selected number of the most abundant pigments (i.e., *chl a, chl b*, α*-car*, β*-car, lut, neo*, and *VAZ*). A handful of studies tended to adopt a metabolomic profiling approach to elucidate carotenoid profiles (Fraser et al., 2000; Gentili et al., 2015, 2019; Wei et al., 2019). Among them, Wei et al. (2019) suggested that accounting for the entire pigment profile rather than the most abundant pigments could better reflect physiological changes in seafood and provide a useful framework for exploring mechanisms underlying pigment composition. A metabolomic profiling approach aims to characterize a pigment extract that is as less altered as possible during sample preparation from sampling until analysis. Yet, techniques that allow for high purity by eliminating the lipid matrix also tend to eliminate chlorophylls and favor conjugated carotenoid hydrolysis (Simonovska et al., 2013). Also, simple extraction of fresh leaf disks either with methanol (Schoefs et al., 1995; Darko et al., 2000), acetone (Airs et al., 2001; Ishida, 2011; Jayaraman et al., 2011), or a mixture of those with water (Garrido et al., 2003) have so far been preferred in ecophysiological studies to avoid alteration of the pigment composition (Supplementary Table 1). However, the solubility and thus the extractability of polar and apolar pigments may differ depending on the polarity of the extraction solvents resulting in an incomplete and selective pigment extraction. In addition, the absence of pre-treatments may allow for the extraction of the free fraction but not bound pigments. While any heat treatment is to be avoided due to the well-documented alteration of pigments at ambient and hot temperatures (Ryan-Stoneham and Tong, 2000), low-temperature pre-treatments (e.g., freeze-drying) offer promising alternatives to enhance pigment extractability.

To date, there is thus a lack of methods investigating simultaneously chlorophylls and carotenoids in complex leaf pigment profiles and minimizing the alteration of the pigments during the preparation and characterization procedure. Therefore, in this study, we developed and validated a method from the sample preparation and extraction to the chromatographic characterization of the pigment extract. We ask whether low-temperature pre-treatment and a sequential extraction with a solvent polarity gradient allow for the recovery of a broader and more representative range of plant leaf pigments than a single-step extraction. In this regard, we showed the benefits of the optimized method to a standardized procedure commonly reported in the literature. We focus on pigments and pigment derivatives that contribute to the leaf's optical properties in the visible range of the light spectrum. We hypothesize that the metabolic profiling approach accounting for low abundance pigments complements what we traditionally learn from the dynamics of the most abundant pigments.

## Materials and methods

### Materials

#### Standards and reagents

Chlorophyll a (≤95 %), chlorophyll b (≤95%), β-carotene (≤95%), and lutein (≤95%) were purchased from Sigma Aldrich (Steinheim, Germany). Standards were prepared in different solvents according to their solubility and stability and later stored in amber vials in a deep freezer at −80°C.

Extraction solvents, acetone (≤99.9%, Carl Roth, Karlsruhe, Germany), isopropanol (≤99.9%, Sigma-Aldrich, Steinheim, Germany), and *n*-hexane (7≤95%, Carl Roth, Karlsruhe, Germany), were GC Ultragrade. Eluents, ethyl acetate (≤99.8%, Merck, Darmstadt, Germany) and methanol (99.9%, Carl Roth, Karlsruhe, Germany), were Hypergrade LC-MS. Water was deionised to >18.2 MΩ/cm^−1^ resistance, using a Milli-Q^®^ Advantage A10 water purification system (Merck, Darmstadt, Germany). Acetic acid (≤60%, Carl Roth, Karlsruhe, Germany) and formic acid (85%, Sigma-Aldrich, Steinheim, Germany) were used as buffers. High-performance liquid chromatography (HPLC) eluents were filtered on glass microfiber filters (Whatman^®^ GF6, Sigma Aldrich, Steinheim, Germany) before use.

#### Leaf samples

Frozen spinach leaves were bought in the local supermarket. The spinach samples were used for the development and validation of the sample preparation, extraction, and liquid chromatography method.

Leaf samples of beech (*Fagus sylvatica L*.) were collected on mature trees located on the campus of the University of Zurich (47°40′ N, 8°55′ E) during the early, middle, and late growing season of 2018. During the early growing season (DOY115–DOY164), young leaves were expending, while the mid-growing season (DOY164–DOY248) corresponded to mature leaves and the late growing season (DOY248–DOY311) to senescing leaves. Three leaf replicates were systematically collected around noon at different positions within the tree canopy to account for variation within a single tree. Immediately after sampling, branches were stored in black plastic bags and stored in polystyrene containers filled with dry ice and transported to a deep freezer at −80°C within an hour. These measures were taken to prevent the degradation of pigments from light and oxygen. These samples were used to demonstrate the applicability of the method to a wide range of samples and the potential of the method to assess intraspecific variation.

### Methods

#### Sample preparation

Within 3 h after the sampling, fresh leaves were individually weighed on an analytical scale (0.01 mg, Satorius Cubis^®^, Germany) to determine the moist weight. Three leaves of the same knot or close to each other were stored at −80°C and analyzed together. Two replicates per branch were prepared. Latest 1 week after sampling, each replicate was freeze-dried overnight (Freeze-dryer Alpha 2–4 LD plus, Christ, Germany). The dry weight of the individual leaves was measured on an analytical scale before the leaves were manually ground in liquid nitrogen with a mortar and pestle.

#### Leaf extraction

Pigments were extracted following a sequential extraction procedure using acetone:water (90:10, v/v), pure acetone, and isopropanol:*n*-hexane (50:50, v/v). Solvents were kept in the freezer at −20°C. Only small volumes of solvent were taken out of the freezer and solvent bottles were immediately returned after use to ensure that pigment extraction was performed below 0°C and to prevent pigment alteration. Fraction 1 was obtained by adding 1 mL of acetone:water directly in the vial containing 20 mg of leaf powder. The solution was agitated using a vortex stirrer and left for 1 min, before being filtered on a 1 μm glass-fiber filter (Macherey-Nagel, Germany) and transferred to a 10 mL amber vial kept cooled in ice. This step was repeated with 1 mL of acetone:water two additional times. The same procedure was repeated with pure acetone and isopropanol:*n*-hexane (50:50, v/v), and the extract was combined in the same vial. The eluate was concentrated under vacuum using a concentrator plus (Vaudaux-Eppendorf, Switzerland), before being transferred to an amber 2 mL vial and redissolved in 250 μL acetone before chromatographic analysis. All steps were performed under subdued light.

#### Chromatographic separation

Chromatographic separation of pigments was carried out with an Agilent 1290 Infinity UHPLC system (Santa Clara, U.S.A) comprised of a binary pump (Agilent G4220A), an autosampler (Agilent G4226A) with the thermostat set to 4°C (Agilent G1330B), a column oven, and a diode array detector (DAD, Agilent VL+ G1315C). The column oven was equipped with one Agilent Poroshell 120 SB-C_18_ (150 mm) with a particle size of 2.7 μm and maintained at 10°C to prevent pigment alteration during the chromatographic separation. C_18_ column was chosen to allow for the separation of pigments covering a wide range of polarity, while the core-shell structure improves the separation of isomers and the peak resolution. A total of 15 μL of standards and samples were injected at a flow rate of 0.5 mL min^−1^, which was kept constant during the analyses. Mobile phase A consisted of purified water buffered with formic acid (98.5:1.5, v/v) to a pH value of 2.5. The mobile phase B consisted of methanol:ethyl acetate (68:32, v/v).

Mobile phase composition, pH, and gradient elution were optimized with a full factorial design of the experiment assessing the pH of eluent A, the composition of eluent B, the initial composition of the mobile phase, and the solvent gradient (Supplementary Figure 2). The optimized chromatographic separation had a total run time of 55 min, which not only allowed for the separation of the most abundant pigments but also minimize the co-elution of pigment derivates. The initial gradient elution yielded 80% eluent B (v/v). After 5 min, the proportion of eluent B in the mobile phase linearly increased to 100% with a ramp of 5%/min. The mobile phase composition was kept constant for 30 min. Re-equilibration time was 15 min between individual measurements.

#### Compound identification and quantification

Compound detection was ensured by a DAD detector at 450 nm for carotenoids and 665 nm for chlorophylls with a split width of 8 nm. Carotenoids with absorption features in the ultra-violet were not considered. The peak integration was performed with the Agilent OpenLAB CDS ChemStation software. Peak identification of main chlorophylls and carotenoids (*chl a, chl b*, β*-car, and lut*) was based on the retention time and absorption spectra of the analytical standards. The %III/II ratio allowed for the identification of additional carotenoids (*neo, vio, zea*, and *ant*). It corresponds to the ratio between the maximum absorption values of the two main peaks absorbing in the visible to which was subtracted the minimum absorption value between the two peaks (Alagoz et al., 2020). Other compound peaks were categorized as chlorophyll and carotenoid derivatives based on a decision tree and the absorption spectra (Supplementary Figure 3). In the case of mixed spectra due to co-elution or a low signal-to-noise ratio that did not allow for the attribution of the absorption spectrum to either chlorophyll or carotenoid derivatives, the compound peaks were labeled as unknown derivatives.

Quantification was based on compound peak areas. Calibration curves from two analytical standards of *chl a* and β*-car* were used to quantify all compounds categorized as chlorophylls and carotenoids (Supplementary Figure 4). The concentrations ranged from 0.3 to 200 μg/mL. The pigment concentration was normalized per dry leaf weight (mg g^−1^). The peak areas of all unknown derivatives were summed and considered as one contribution.

#### Statistical evaluation

The statistical evaluation was performed with the R studio software (R Core Team, 2020), with the packages *FrF2, DoE.base*, and *DoE.wrapper* for the design of the experiment and *tidyvers*e, *dplyr*, and *ggplot2* for other analyses.

#### Method validation

**Figure 1:**
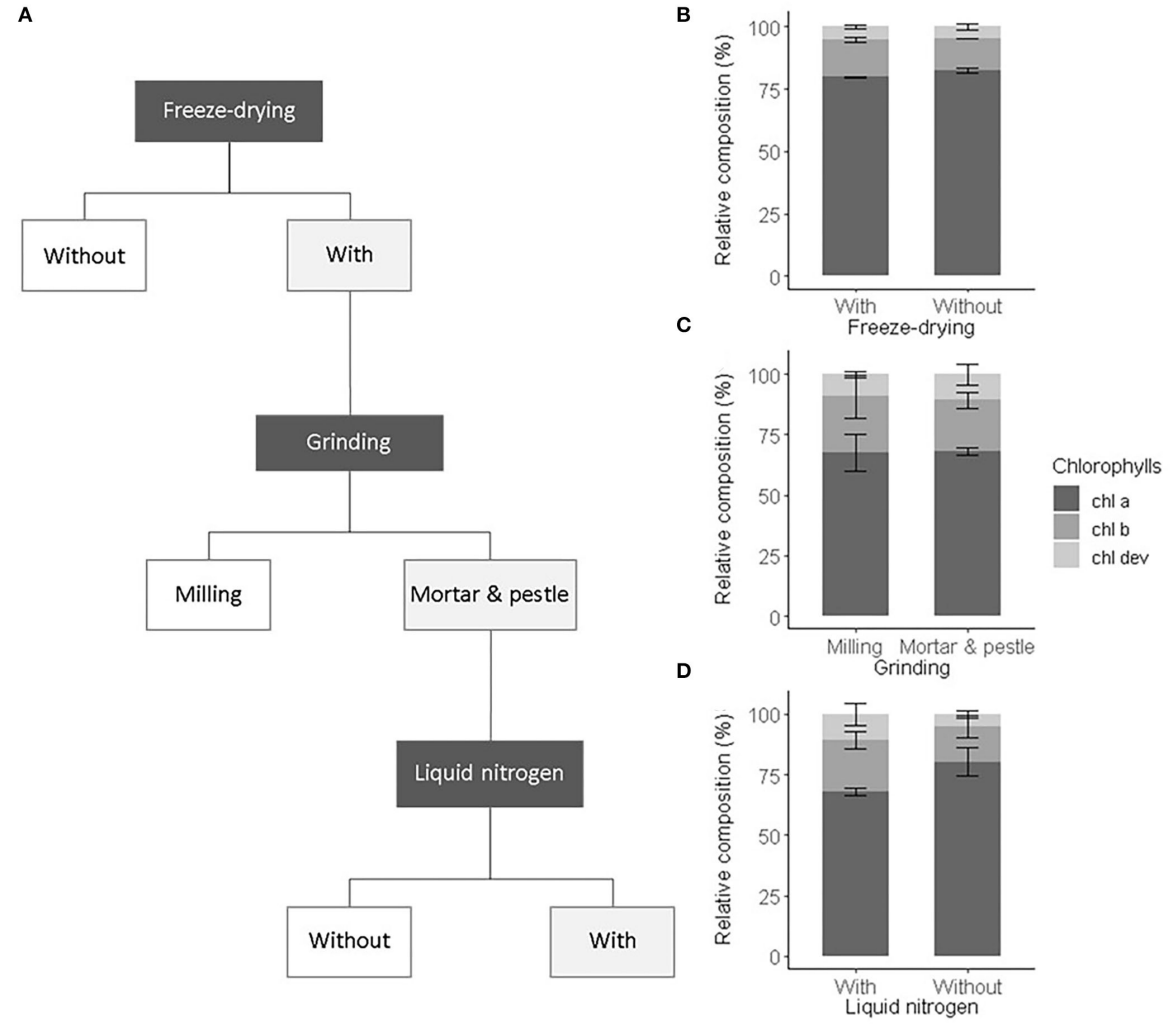
Sample preparation procedure and its impact on the chlorophyll composition. **(A)** Selected preparation steps (light gray boxes) and the relative composition of chlorophylls including chl a (dark gray), chl b (gray), and chl dev (light gray) associated with **(B)** freeze drying, **(C)** grinding, **(D)** with or without liquid nitrogen. Error bars correspond to the standard deviation (*n* = 3).

As an initial test to validate the sample preparation procedure, we compared the influence of sample preparation steps commonly performed in ecophysiological studies on the characterized pigment profiles. Also, we verified that freeze-drying and manual milling did not alter the pigment recovery and composition (i.e., ratios of *chl a, chl b*, and *chl dev*). We first compared the pigment composition obtained from frozen spinach leaf extracts and freeze-dried extracts of the same leaf samples. We then compared the effect of automatic milling, manual milling with liquid nitrogen to no milling on pigment composition and recovery (Figure 1A). The same ground leaf sample was used to test all preparation procedures. Three analytical replicates were prepared. We applied a one-way ANOVA test on the total pigment concentration and on *chl a, chl b*, and *car* concentrations to assess the recovery and on the number of detected peaks and *chl a:chl b* and *chl a:chl dev* ratios to validate the composition.

The extraction procedure was validated for a selection of five samples of spinach leaves. The extracts obtained after each solvent were collected in separate vials, separately analyzed, and compared to the corresponding extract of the control procedure. In the control procedure, the same samples were extracted using a unique solvent mixture of acetone:water (90:10, v/v) for all three fractions. We looked at the absence/presence of compound peaks and applied a *t-*test to compare the overall and individual fraction yields. Repeatability was tested on five analytical replicates.

The validation of the chromatographic separation was performed according to the recommendations of Stöckl et al. (2009) and Raposo and Ibelli-Bianco (2020). We tested five performance parameters listed in Table 1 (Guy, 2014; ISO/IEC Guide 99, 2007). The selectivity was determined for the main pigments *chl a, chl b*, and β*-car* by calculating the selectivity factor between the main pigments and the neighboring peaks. The application range was defined based on the calibration curve including six concentrations measured in two replicates over 2 days (1r × 2d) and validated with an ANOVA lack-of-fit test (Stöckl et al., 2009). A calibration curve was established for each individual analytical standard of *chl a, chl b*, and β*-car*, dissolved in pure acetone (Supplementary Figure 4). The accuracy was defined as the total error including the precision (random error) and the trueness (systematic error). The intra-day and inter-day precision were assessed by measuring a spinach and beech extract twice a day for five consecutive days. The trueness was measured at the concentrations of *chl a* standard for three replicates with an acceptance criterion of < 5%. The limit of detection (LOD) and limit of quantification (LOQ) were set at a signal-to-noise ratio of 3 and 6, respectively (Guy, 2014; Stöckl et al., 2009).

**Table 1:** Tested performance parameters of the chromatographic separation.

**Performance parameters**	**Protocol**	**Acceptance criteria**	**Samples**	**Compounds**	**Results**
	*replicates x days*				
**Selectivity**	Full factorial DoE	Selectivity factor >1.3	Spinach Beech	*chl a, chl b, β-car*	*chl a*: 1.4 *chl b*: 1.5 *β-car*: 1.2
**Application range**	6 concentrations 1r x 2d	ANOVA lack-of-fit test	Standards	*chl a, β-car*	*P* < 0.05 (Supplementary Figure 4)
**Intra- and inter- precision**	3r x 5d	≤10%	Spinach Beech	*chl a, chl b, chl dev, β-car, car dev*	≤7.8%
**Trueness**	2r x 5d	≤5%	Standards	*chl a*	≤1%
**LOD**	none	Signal-to-noise ratio of 3	Blank	*chl dev, car dev*	
**LOQ**	none	Signal-to-noise ratio of 6	Standards Spinach	*chl dev, car dev*	

## Results and discussion

### Method development and validation

#### Sample preparation

We tested the influence of the preparation steps, i.e., freeze-drying and grinding, on the extraction yield and the relative pigment composition (Figure 1A).

Freeze-drying was carried out to remove the presence of moisture in leaf samples and prevent pigment alteration during storage (Çinar, 2004; Thamkaew et al., 2021). Freeze-drying tended to increase the extraction yield, though not significantly (ANOVA, F_1,4_ = 4.53, *p* = 0.167). The sublimation of frozen water crystals under vacuum contributes to break cell walls and membranes, facilitating solvent accessibility and the release of pigments from the protein matrix (Thamkaew et al., 2021). In this sense, higher concentrations of *chl a* and *chl b* in freeze-dried extracts than in fresh frozen extracts of mint leaves were found (Rubinskiene et al., 2015). However, the opposite was also observed in extracts of algae (Amorim et al., 2020). Also, our result confirmed that the effect of freeze-drying on the extraction yield is species-specific and depends on the leaf structure and composition. Among drying procedures, freeze-drying is known to best preserve pigments (Thamkaew et al., 2021). This was verified for our leaf samples, whose pigment composition was statistically identical among fresh frozen and freeze-dried leaf extracts (ANOVA, F_1,4_ = 2, *p* > 0.1) (Figure 1B). Nonetheless, freeze-drying increases sample porosity and thus higher surfaces are potentially exposed to oxidation and moisture reabsorption than in fresh frozen leaves (An-Erl King et al., 2001). Besides, pigments released from the protein matrix may be more sensitive to heat, photochemical, and enzymatic degradation (Hu et al., 2013; Thamkaew et al., 2021). Also, as a precautionary measure, freeze-dried leaf samples were stored in sealed black bags at −80°C and later handled under subdued light and below 0°C.

Freeze-dried leaf samples were ground to further break the cell walls and membranes, facilitate solvent accessibility, and eventually improve the pigment extraction yield in comparison to the one obtained from commonly non-ground fresh leaf disks (García-Plazaola and Becerril, 2000b; García-Plazaola et al., 2008; Scartazza et al., 2016). The use of a mill ball or a mortar and pestle to grind the leaf samples led to similar extraction yield and relative pigment composition (ANOVA, F_1,4_ = 3, *p* > 0.1) (Figure 1C). Automatic milling using, e.g., ball mills, is widely used to produce homogeneous leaf sample powder. However, the repeated friction of the sample with the ball of a horizontal mill may heat the sample and alter its composition, if the milling device cannot be cooled or the duration of the milling process is too long (Markert, 1995). In this study, the settings of the ball mill were chosen to prevent pigment alteration due to over-heating as evidenced by the pigment composition that is comparable to the one obtained after manual grinding with liquid nitrogen (Figure 1C). However, more material was lost on the wall of the beaker of the ball mill than within the mortar. In addition, the cleaning of the beaker of the ball mill (necessary to avoid contamination) was more time-consuming than the one of the mortar and pestle, and consequently manual grinding was preferred. The use of a mortar and pestle with liquid nitrogen resulted in a finer and more homogenous powder than without liquid nitrogen or with a ball mill, as also shown by Gomes et al. (2011). This eventually improved the extraction of *chl dev* (ANOVA, F_1_,_4_ = 4.8, *p* = 0.093) and decreased the relative contribution of *chl a* (ANOVA, F_1,4_ = 13.9, *p* = 0.022) (Figure 1D). As the overall yield was greater with than without liquid nitrogen and as the manual grinding was performed under subdued light with liquid nitrogen, we are confident that the greater proportion of *chl dev* corresponds to the better extraction of low abundant pigment compounds using liquid nitrogen and not denaturized pigments resulting from potential preparation artifacts (Hu et al., 2013).

#### Extraction

**Figure 2:**
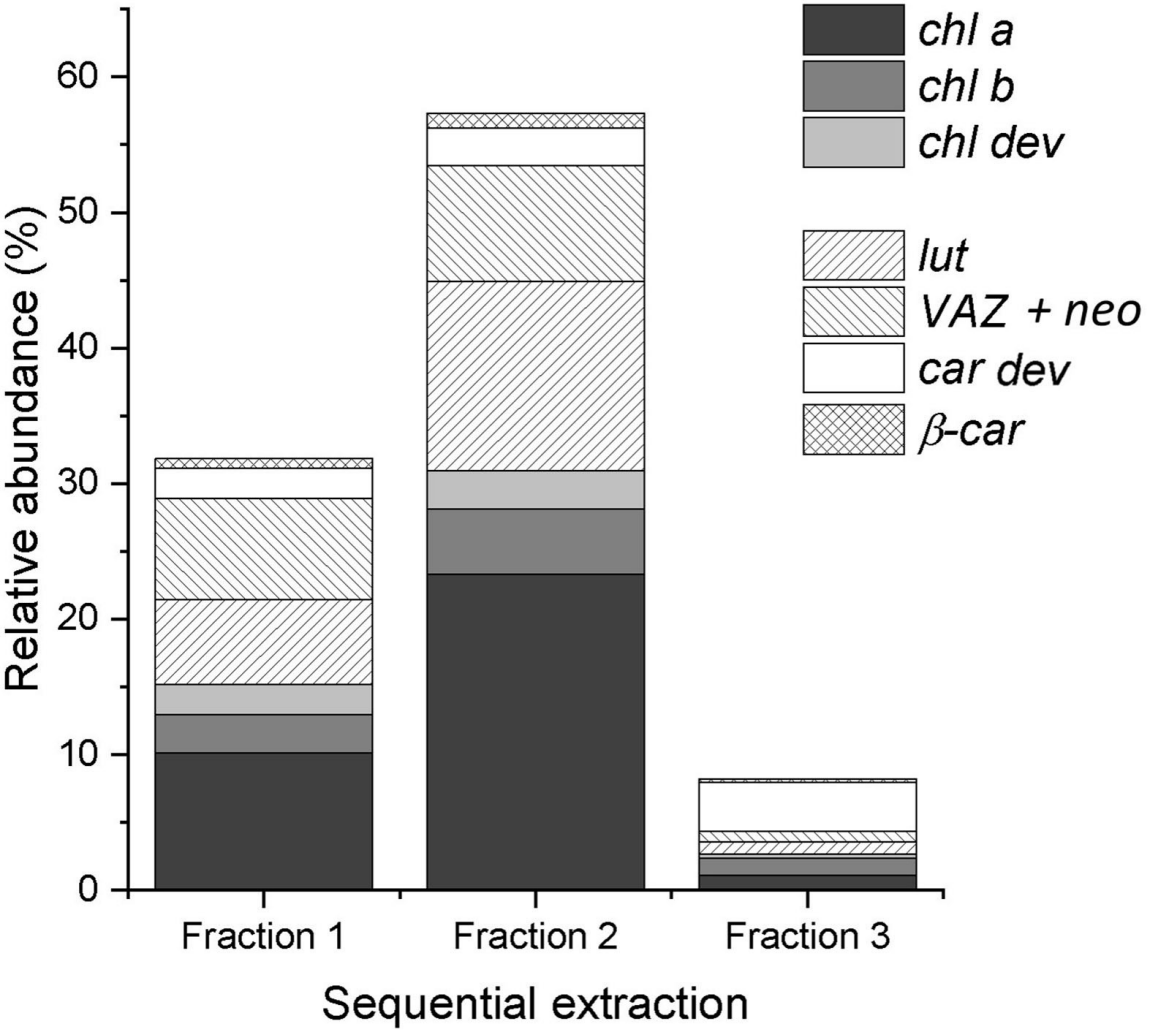
Relative abundance of chlorophylls (chl a, chl b, and chl dev) and carotenoids (β-car and car dev) in each fraction of the sequential extraction of a spinach sample. The sum of the three fractions equals 100%.

On average, the sequential extraction with a solvent polarity gradient allowed for the extraction of three times more compounds than using acetone:water (90:10, v/v) as a unique solvent (Figure 2). Up to 62 chromatographically resolved pigment compounds (potentially comprising isomers), including 28 *chls*, 24 *car dev*, and 10 unknown compounds resulted from the sequential extraction of spinach leaves (Table 2). Apart from 10 compounds found only in fraction 1 (acetone:water), all observed compounds were present in fraction 2 (pure acetone). The last extraction step (isopropanol:*n*-hexane) improved on average by 16 ± 6% (SD) the extraction yield of compounds present in all fractions (*n* = 25 compounds) and by 29 ± 7% the extraction of apolar compounds found in fraction 2 and 3 only (*n* = 6 compounds). The extraction yield achieved by sequential extraction with the solvent polarity gradient was on average 40% greater than with a repeated extraction using acetone:water (90:10 v/v) as a unique solvent. Individual fractions respectively contribute 33% (Fraction 1), 58% (Fraction 2), and 9% (Fraction 3) to the extraction yield of the sequential extraction with the chosen solvent polarity gradient (Figure 2A). Fractions 2 and 3 contribute to 25, 30, 42, and 61% of the extraction yield of *chl a, chl b*, β*-car*, and xanthophylls (i.e., lut + neo + VAZ), respectively.

**Table 2:** Table indexing retention times, pigment groups, absorbance maxima of each peak, and %III/II ratios that were observed in the chromatogram of spinach leaves.

**Peak nb**	**RT**	**Compounds**	**Absorption maxima**			**III/II**	**Spectrum**
	***min***		***λ (nm)***			***%***	**quality**
1	5.91	chl dev	468	600	654	NA	+
2	7.86	chl dev	468		652	NA	-
3	8.95	unknown	-			NA	–
4	9.70	chl dev	434	620	668	NA	++
5	10.40	unknown	-			NA	–
6	11.15	unknown	-			NA	–
7	12.18	car dev	440			NA	-
8	12.45	chl dev	434		668	NA	-
9	12.87	car dev	440	472		NA	+
10	13.72	car dev	438	472		NA	-
11	14.39	car dev	484			NA	-
12	14.87	car dev	434			NA	++
13	15.02	car dev	432	458		28.7	+
14	15.49	car dev	440	470		94.3	++
15	15.73	chl dev	420		666	NA	-
16	16.37	9-*cis*-violaxanthin (VAZ)	436	464		87.4	++
17	16.60	car dev	444	472		53.8	-
18	16.95	car dev	434	466		92.4	-
19	17.06	chl dev	-			NA	–
20	17.46	All-*trans*-neoxanthin (*neo*)	440	470		91.8	++
21	17.76	unknown	-			NA	–
22	18.16	car dev	420	448		91.9	+
23	18.40	car dev	420	450		NA	+
24	18.70	unknown	-			NA	–
25	19.10	unknown	-			NA	–
26	19.26	unknown	-			NA	–
27	19.62	car dev	442	468		63.8	+
28	20.05	car dev	436	462		61.9	+
29	20.25	car dev	436	462		NA	-
30	21.18	car dev	446			NA	-
31	21.44	lutein (lut)	446	474		63.0	++
32	22.22	car dev	-			NA	–
33	22.87	chl dev	420		666	NA	–
34	23.13	antheraxanthin (VAZ)	446	474		57.7	+
35	23.25	all-*trans*-zeazanthin (VAZ)	450	478		30.9	+
36	23.46	chl dev	454	580	634	NA	++
37	23.67	unknown	454			NA	–
38	23.85	chl dev	454		632	NA	-
39	23.90	unknown	454			NA	-
40	24.16	chl dev	454		644	NA	-
41	24.45	chl dev	434		660	NA	-
42	24.65	chl dev	436		660	NA	-
43	24.82	chl dev	468	600	652	NA	++
44	25.22	chl dev	470		654	NA	-
45	25.36	chl dev	434		658	NA	-
46	25.59	chl dev	420		664	NA	-
47	26.49	chlorophyll b (*chlb*)	468	602	652	NA	++
48	26.83	chl dev	420		656	NA	-
49	27.15	chl dev	468	602	652	NA	++
50	27.51	chl dev	434		668	NA	–
51	27.87	chl dev	428		666	NA	–
52	28.05	chl dev			664	NA	–
53	28.45	chl dev	432	620	666	NA	++
54	29.17	chl dev	430	618	664	NA	+
55	29.47	chl dev			668	NA	–
56	30.31	unknown	474			NA	-
57	30.73	chl dev			668	NA	–
58	31.24	chlorophyll a (*chla*)	432	618	666	NA	++
59	33.07	chl dev	432	618	666	NA	++
60	43.94	car dev	-			NA	–
61	52.21	α-carotene (*α-car*)	446	472		61.3	+
62	53.32	β-carotene (*β-car*)	456	476		13.6	+

Aqueous and pure acetone have been equally used and preferred among other solvents, such as ethanol, dimethylformamide, and dimethyl sulfoxide for the extraction of photosynthetic pigments of leaf samples (Supplementary Table 1). Our results confirmed that aqueous and pure acetone allow for the extraction of the main pigments (including *chl a, chl b*, lut, neo, VAZ, and β*-car*) enabling a fast screening of the pigment profile. However, aqueous acetone and pure acetone did not perform equally at extracting pigment derivatives. Pure acetone allowed for the extraction of most of the pigment derivatives. Nevertheless, highly polar and apolar pigment compounds were not present in the acetone fraction. Highly polar chlorophylls and carotenoids were best extracted with acetone:water (90:10, v/v), despite the fact that lyophilized leaf samples were expected to have a better affinity with acetone than aqueous acetone mixture (Lichtenthaler, 1987). Besides, the solubility of carotenoids in pure acetone decreases with decreasing compound polarity, as well as the ability of acetone to break carotenoid–protein complexes (Simonovska et al., 2013). As highlighted by Simonovska et al. (2013), the ability of the solvent to break down pigment–protein complexes matters more for the extraction efficiency than for the solubility of the compounds in the solvent mixture, especially if the concentration of pigments in the leaf sample is low. Extended extraction times were commonly described in the literature (up to 48 h) to compensate for the weak extraction power of acetone but only the most abundant pigments were targeted (Garrido et al., 2003; Hu et al., 2013). Besides, pigment recovery and composition were shown to differ among extraction solvents, with an extraction carried for 12 h at 4°C, the enzyme chlorophyllase was shown to catalyze the degradation of *chls* into chlorophyllides in aqueous acetone but not in pure acetone (Hu et al., 2013). Those observations validated our first hypothesis that a single extraction with (aqueous or pure) acetone led to a biased representation of the leaf pigment profile due to the polarity of the extraction solvent and its interaction with the leaf constituents that potentially lead to extraction artifacts. Frequently used methods (see Supplementary Table 1) might thus not be able to reliably characterize the majority of the pigment profile. The presented method relied on sequential extraction with a solvent polarity gradient. By gradually decreasing the polarity of the solvent, we succeeded in extracting apolar pigment derivatives and significantly increased the extraction yield of all compounds. Isopropanol:*n*-hexane showed a greater ability to extract compounds embedded in pigment–protein complexes than acetone, which enabled to extract a wide range of pigments in a short time. The short extraction time and temperatures below 0°C aimed to limit pigment alteration.

#### Chromatography

**Figure 3:**
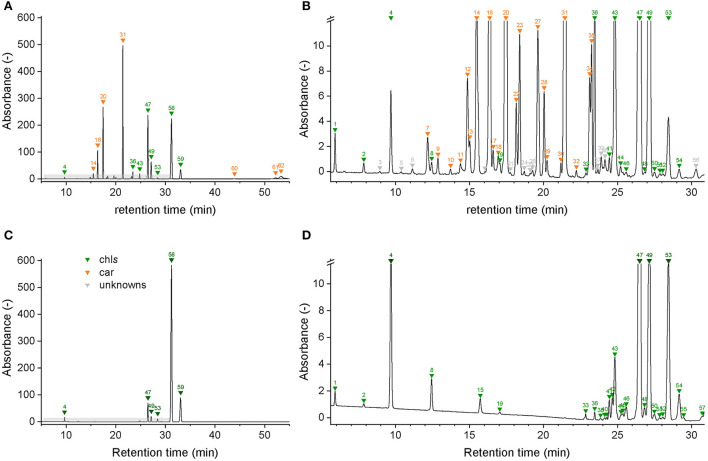
Chromatograms of a spinach sample at 450 nm **(A,B)** and at 665 nm **(C,D)**. Individual compounds are listed in Table 2.

Chromatographic separation of the main pigments *chl a, chl b*, and *car*, and related pigment derivatives that are partially present in low abundance was achieved within 55 min (Figure 3). Pigment derivatives represented on average 95% of the detected peaks but less than 34% of the integrated peak area counts. Up to 62 chromatographically resolved pigments (potentially comprising isomers), including 28 chlorophylls, 24 carotenoids, and 10 unknown pigments resulted from the sequential extraction of spinach leaves (*Spinacia oleracea*) (Figure 3; Table 2). We obtained similar chromatograms for deciduous tree species, including beech (*Fagus sylvatica L*.), lime (*Tilia cordata Mill*.), and maple (*Acer pseudoplatanus L*.) (Supplementary Figure 5). The pigment derivatives categorized as unknown were either below the limit of identification or co-eluted with other compounds. Nevertheless, the latter were few and a large number of photosynthetic pigments were successfully categorized based on their absorption spectra (Supplementary Figures 6, 7). The number of categorized compounds exceeded what was previously achieved in a similar run time for spinach [up to 26 pigments, (Schwartz et al., 1981; Gokmen et al., 2002; Kidmose et al., 2005; Stinco et al., 2014)] or beech [*chl a, chl b*, and up to 11 *car dev* (García-Plazaola and Becerril, 1999; Junker and Ensminger, 2016)]. Low abundance pigment derivatives were mostly neglected in previous methods. Highly concentrated extracts and the use of a chromatographic column with a poroshell structure, which improved peak shape compared to a column packed with porous particles, contributed to an increase in the number of detected pigments.

The influence of four parameters, i.e., the pH of the eluent A, the polarity of the eluent B, the initial eluent ratio, and the eluent gradient, on the chromatographic separation was assessed by carrying out a full factorial experimental design (Supplementary Figure 2). pH mostly affected the chromatographic separation (< 3, F_1,11_ = 5.185, *p* < 0.05). Acidic pH increased the number of detected peaks by reducing co-elution and improving peak separation. However, *chls* degrade into pheophytins and *car dev* isomerize more easily at acidic pH than at neutral and basic pH (Gunawan and Barringer, 2000; Meléndez-Martínez et al., 2010). Nevertheless, alteration of pigments is expected to happen slower at low temperatures (Ryan-Stoneham and Tong, 2000). To validate the optimum pH, an analytical standard of *chl a* was run at different pH values. A pH of 3 allowed for the best selectivity, a good reproducibility, and a limited alteration of the analytical standard of *chl a*. A pH comprised between 3 and 4 was also considered optimal to characterize *chl dev* as chlorophyllides and pheophytins. The solvent gradient (F_1,11_ = 3.385, *p* = 0.1) and to a lesser extent the composition of eluent B and its proportion in the initial mobile phase (F_1,11_ = 2.032, *p* = 0.182) also affected the chromatographic separation (Supplementary Figure 2). Most compounds appeared during the isocratic phase (100% of eluent B). The run time was thus optimized by decreasing the polarity of eluent B and increasing the proportion of eluent B in the initial mobile phase.

Method validation was performed by evaluating the selectivity, application range, limits of detection and quantification (LODs and LOQs), precision (inter-day and intra-day precision), and trueness of the main pigments and pigment derivatives (Table 1). The main pigments (*chl a, chl b*, and β*-car*) as well as some pigment derivatives presented a selectivity >1.3. Co-elution of some pigment derivatives was still observed. However, it was considered sufficient for sample screening, as the low abundance of co-eluted pigment derivatives made the identification challenging for individual pigment derivatives that were eventually investigated as one group. The application range was validated between 0.3 and 2 μg mL^−1^ with an increasing residual error at low concentrations (Supplementary Figure 4). The intra- and inter-day precision and the trueness were respectively below the targeted threshold. Also, with a large number of characterized pigments and acceptable precision for ecophysiological studies, the developed method offers the opportunity to investigate the dynamics of the main photosynthetic pigments and their derivatives.

### Application

To demonstrate the applicability of the proposed analytical method, we characterized the pigment profile of leaves originating from one individual beech tree. As it is well known that the leaf pigment composition changes during the growing season (Scartazza et al., 2016; Kraj and Zarek, 2021), we tested and validated our method on leaves sampled in the early, middle, and late growing season. We compared the pigment concentration and composition obtained after a single step and sequential extraction using always the optimized chromatographic procedure. The concentration of total chlorophyll (*chl tot*) was respectively comprised between 0.3 and 2.6 μg *chl a* eq.g^−1^d.w., and 1.8 and 45.2 μg *chl a* eq.g^−1^d.w. over the course of the growing season (Supplementary Figures 8A,B). The sequential extraction increased on average by 10 times the recovery in *chl tot, chl a an chl b* and by up to 40 times the recovery in *chl dev* compared to a single-step extraction. The range of concentrations of *chl tot* obtained with a single-step extraction was comparable to the concentrations in beech leaves reported in the literature using similar extraction methods (Supplementary Table 1) (Herbinger et al., 2007; Scartazza et al., 2016; Primka and Smith, 2019; Kraj and Zarek, 2021) and fell into the expected range of concentrations (1.2–15.6 μmol.g^−1^d.w.) defined by Esteban et al. (2015) in a meta-analysis including 809 plant species. The range of concentrations obtained with a sequential extraction, however, exceeded this expected range of concentrations but remained close to the concentration range (11.1–44 μmol.g^−1^d.w.) suggested by Antal et al. (2013). The concentration of total carotenoids (*car tot*) obtained with a sequential extraction was comprised between 8.1 and 64.3 μg *chl a* eq.g^−1^d.w., i.e., 12 times more than with a single-step extraction (0.7 and 7.4 μg *chl a* eq.g^−1^d.w.) (Supplementary Figures 8C,D). *car tot: chl tot* was on average of 2.5 ± 0.8. It did not statistically differ from the extraction method (F_1,38_ = 0.428, *p* = 0.517) and was in the same order as the ratios reported in the literature (García-Plazaola and Becerril, 2000a; Herbinger et al., 2007; Esteban et al., 2015). The range of concentration consequently depends on the preparation and extraction methods. The greater recovery of pigments and greater contribution of pigment derivatives after a sequential extraction than a single extraction resulted in a wider range of concentrations that helped to better capture seasonal dynamics and variation within the tree crown.

**Figure 4:**
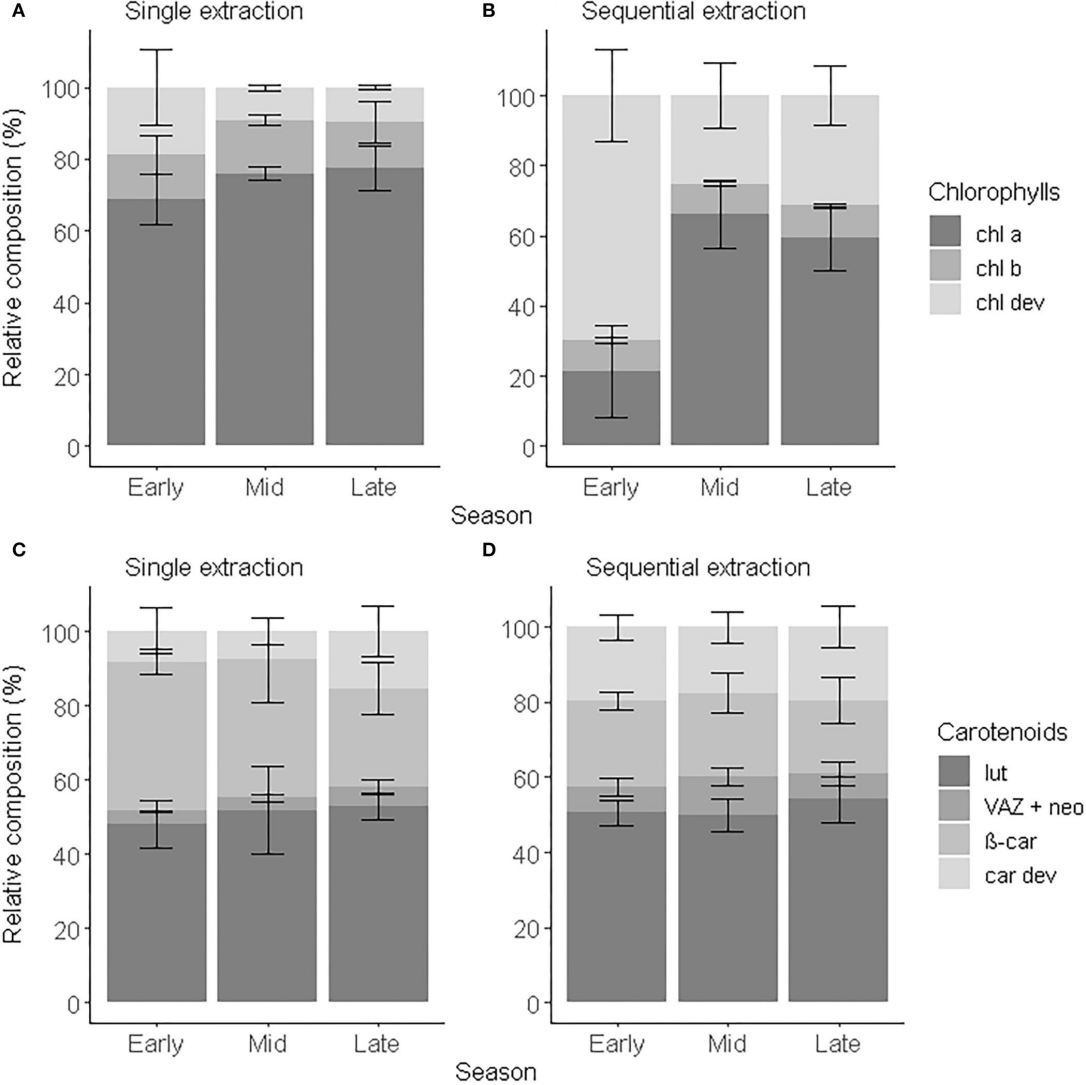
Relative composition of chlorophylls **(A,B)** and carotenoids **(C,D)** of beech leaves in early (May to June), mid (July to August), and late (September to November) growing season 2018 obtained after a single extraction **(A,C)** and a sequential extraction **(B,D)**. Error bars correspond to standard deviations (*n* = 4).

Variation of *chl a* and *chl b* within the crown was observed between the sun and shade leaves (Herbinger et al., 2007; Wieser et al., 2003) or along a vertical canopy gradient (Niinemets et al., 2015; Scartazza et al., 2016), which brought new insights about the photosynthetic activity of a tree. Similarly, *chl a* and *chl b* are used as proxies to investigate seasonal dynamics and leaf senescence (García-Plazaola and Becerril, 1999; Kraj and Zarek, 2021). However, Esteban et al. (2015) showed that *chl a:chl b* tended to vary over a restricted range and showed little responsiveness to environmental stress, such as drought and ozone exposure. Here, we showed that *chl dev* concentration varied across the season and within the tree crown (i.e., standard deviation) offering an additional layer of understanding of the pigment dynamics. *chl dev:chl tot* was significantly greater and *chl a*+*b:chl tot* lower (ANOVA, F_1,16_ = 8.34, *p* < 0.001) after a sequential extraction than a single-step extraction (Figures 4A,B). Irrespective of the extraction method, *chl dev:chl tot* was highest in the early growing season (single-step: 0.2 ± 0.1, sequential: 0.7 ± 0.1) and tended to be at its lowest in the middle of the growing season (single-step: 0.09 ±0.01, sequential: 0.3 ± 0.1). The opposite was observed for *chl a*. Independently of the extraction method, *chl b:chl tot* remained constant over the entire growing season (single-step: 0.13 ± 0.01, sequential: 0.090 ± 0.002, F_1,16_ = 2.90, *p* = 0.108). The standard deviation indicates the variability in pigment composition within an individual tree among different sampling spots. The pigment profile obtained with a sequential extraction tended to capture the most variability. This variability is mostly driven by *chl a:chl tot* (cv = 30%) and *chl dev:chl tot* (cv = 28%) and to a lesser extent *by chl b:chl tot* (8%). The variability in *chl a:chl tot* within an individual tree is maximum in the early season and minimal in the mid-season and reversely for *chl dev:chl tot*. There was no variability observed for *chl b:chl tot* within an individual tree across the season. Pigment derivatives are precursors and degradation products of the major pigments and thus can inform the renewal of *chl a* and *chl b*, especially when the *chl a:chl b* ratio tends to be conserved in the mid-growing season (Polle et al., 2001; Šprtová et al., 2003; Hmimina et al., 2015). Change in color was shown to not be a good indicator of chlorophyll degradation in leaves (Primka and Smith, 2019), potentially due to the contribution of *chl dev* to the optical signal (Kraj, 2015). Regardless of the extraction method, we unsurprisingly observed an increase in *car tot* with time, notably due to leaf senescence as previously reported for *Fagus sylvatica* (García-Plazaola and Becerril, 2001) and other deciduous tree species (Czeczuga, 1986). The carotenoid distribution did not statistically differ over time because of high variability (i.e., the standard deviation) within each phenological stage (Figures 4C,D). Nevertheless, the proportion of *VAZ* + *neo* tended to decrease with time, while the proportion of *car dev* increased. This would be coherent with the esterification of xanthophylls commonly observed in stressed and senescent leaves (García-Plazaola and Becerril, 2001). The contribution of *car dev* to *car tot* was on average two times greater with a sequential extraction than (16.5–29.7%) with a single-step extraction (7.6–15.4%) and accounted for more individual compounds. Therefore, characterizing pigment derivatives enables to better describe the leaf optical properties which represents an opportunity to calibrate optical sensors. In addition, monitoring pigment derivatives offer the opportunity to further explore seasonal dynamics and variation within the tree crown, in relation to photosynthesis activity and microclimate.

## Conclusion

In this study, we described a new method for the simultaneous characterization of leaf chlorophylls, carotenoids, and their derivatives (including isomers). Alterations of the pigment profile inherent to the preparation, extraction, and chromatographic separation were assessed and lessened using precautionary measures that guaranteed a good reproducibility of the results. The sequential extraction allowed for the recovery of the main pigments and pigment derivatives over a broader range of polarity in comparison with a single extraction commonly used in the literature. The first intention of the method was to characterize pigments contributing to the leaf optical properties in the visible range of the light spectrum more exhaustively to calibrate optical sensors. Nevertheless, the fingerprint of leaf pigments can serve other research questions related to forest tree physiology. Also, we used a standard HPLC-DAD system with the aim to provide a method accessible to a wide range of users and suitable for diverse applications. The chromatographic separation was validated for leaves of common European deciduous tree species with good precision and application range for ecophysiological applications. We showed in a case study that accounting for pigment derivatives improved the characterization of intraspecific variation within an individual tree over one growing season.

## Data availability statement

The original contributions presented in the study are included in the article/Supplementary materials, further inquiries can be directed to the corresponding author/s.

## Author contributions

FP and GW: conceptualization, methodology, and writing—review and editing. FP: data curation, investigation, formal analysis, visualization, and writing—original draft. GW: funding acquisition, resource, project administration, and supervision. Both authors contributed to the article and approved the submitted version.

## Funding

This study was supported by the University of Zurich including the Swiss National Science Foundation Project 157778 that funded the HPLC system and the University Research Priority Program on Global Change and Biodiversity.

## Conflict of interest

The authors declare that the research was conducted in the absence of any commercial or financial relationships that could be construed as a potential conflict of interest.

## Publisher's note

All claims expressed in this article are solely those of the authors and do not necessarily represent those of their affiliated organizations, or those of the publisher, the editors and the reviewers. Any product that may be evaluated in this article, or claim that may be made by its manufacturer, is not guaranteed or endorsed by the publisher.

## Abbreviations

ant: antheraxanthin
car: carotenoids
α-car: alpha-carotene
β-car: beta-carotene
car dev: carotenoid derivatives
chls: chlorophylls
chl a: chlorophyll a
chl a eq.: chlorophyl a equivalent
chl b: chlorophyll b
chl dev: chlorophyll derivatives
chl tot: total chlorophyll
cv: coefficient of variation
DAD: Diode Array Detector
d.w.: dry weight
HPLC: High Performance Liquid Chromatography
LOD: Limit Of Detection
LOQ: Limit Of Quantification
Lut: lutein
Neo: neoxanthin
RSD: Relative Standard Deviation
UHPLC: Ultra High Performance Liquid Chromatography
VAZ: xanthophyll cycle
vio: violaxanthin
v/v: volume per volume
xan: xanthophyll
zea: zeaxanthin.
