# Supplementary material for: Characterization of complex photosynthetic pigment profiles in European deciduous tree leaves by sequential extraction and reversed-phase high-performance liquid chromatography
Source: Front Plant Sci. 2022 Oct 12;13:957606. doi: 10.3389/fpls.2022.957606 (PMC9605812; doi:10.3389/fpls.2022.957606)
Supplement: Supplementary file 1 [file Data_Sheet_1.docx]

Supplementary Material

| **Chlorophylls**  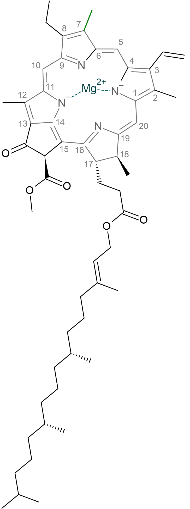    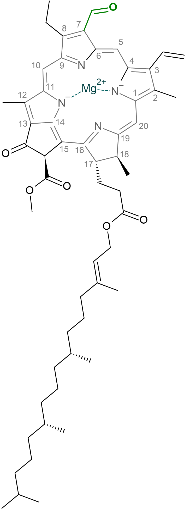 a. Chlorophyll a    b. Chlorophyll b |  | **Carotenoids** |  |
| --- | --- | --- | --- |
|  |  | **Carotenes** | |
|  |  | 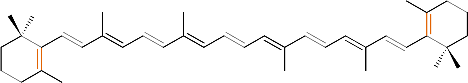  c. β-carotene | 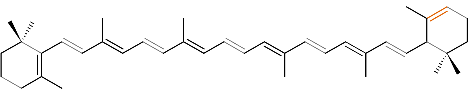  d. α-carotene |
|  |  | **Xanthophylls** | |
|  |  | 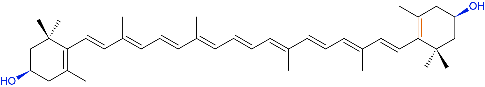  e. Zeanxanthin  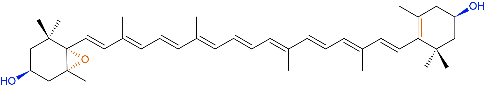  f. Antheraxanthin  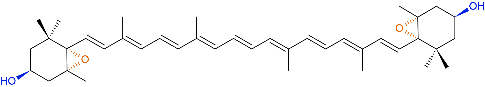  g. Violoxanthin  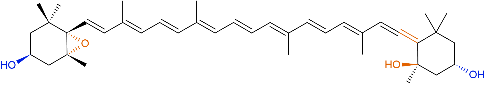  h. Neoxanthin | 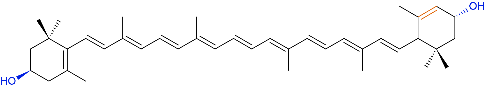  i. Lutein |

**Supplementary Figure 1. Main chlorophylls and carotenoids present in tree leaves.** Chlorophylls (a-b) consist of a porphyrin skeleton chelating a magnesium ion in the centre. Chlorophyll a and chlorophyll b are non-polar and esterified forms of chlorophyll, characterized by a vinylic group, a methyl ester, and a phytyl chain. Chlorophyll a and chlorophyll b differ by the substituent on carbon 7, respectively a methyl and aldehydic group, which confers to chlorophyll a and b distinct light absorption properties. Carotenes are tetraterpenes consisting of eight isoprene units (bold). β-carotene (c) and α-carotene (d) are the two primary isomers. Xanthophylls are oxygenated tetraterpene derivatives. All green plants have in common five xanthophylls: Zeaxanthin (e), antheraxanthin (f), violaxanthin (g), neoxanthin (h), and lutein (i).

**Supplementary Figure 2. Main effects for the response (a) number of peaks and (b) resolution score.** The design of experiment allows for the assessment of four variables **(A)** the pH of eluent A (water buffered with acetic acid), **(B)** the composition of the eluent B (methanol: ethyl acetate), **(C)** the initial composition of the mobile phase (eluent A: eluent B ratio), and **(D)** the gradient elution which levels are above listed (c). Two responses were evaluated: (a) The number of peaks observed at 450 nm and 665 nm and (b) the resolution score. The horizontal line represents the mean response, while the dots indicate the response at levels -1 and 1 for each variable. The effect size is symbolized by the length of the segment. The significance of the main effects and interactions was tested using ANOVA. The number of peaks significantly increased at low pH (A, F_11_=5.185, p < 0.05) and fast gradient elution (D, F_11_=3.385, p>0.1). All other effects including interactions were found nonsignificant. Nevertheless, low pH (A, F_11_=2.156, p=0.170) and low amount of eluent A in the initial composition of the mobile phase (C, F_11_=2.032, p=0.182) tend to improve the separation.Consequently, the composition of the eluent B was kept at level 0 (MeOH: EtAc, 68:32, v/v), while level 1 was selected for the gradient elution (4%/min) and the initial composition of the mobile phase (A:B, 20:80, v/v) in order to maximize the number of peaks and their selectivity. Eluent A was eventually buffered at pH =2.5 to maximise the peak separation while still ensuring a good selectivity and avoiding pigment alteration.


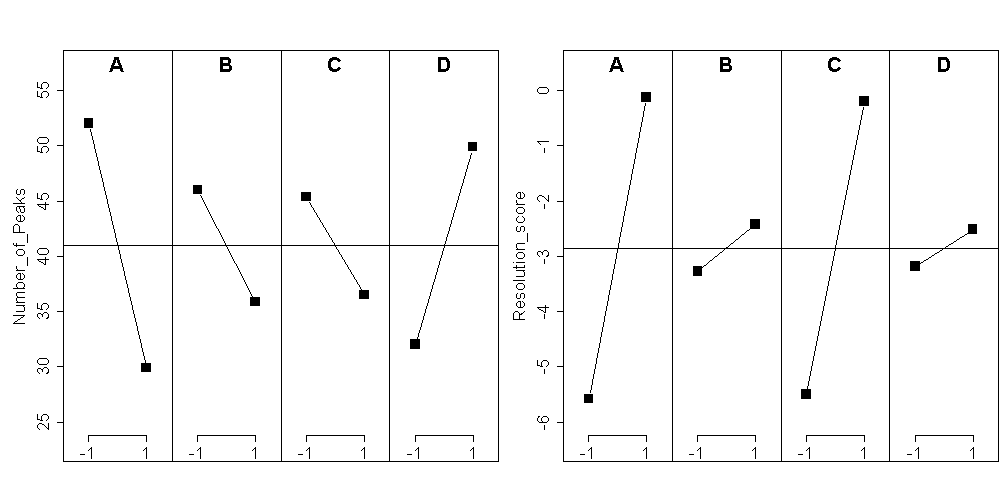


**a) b)**

**c)**

**
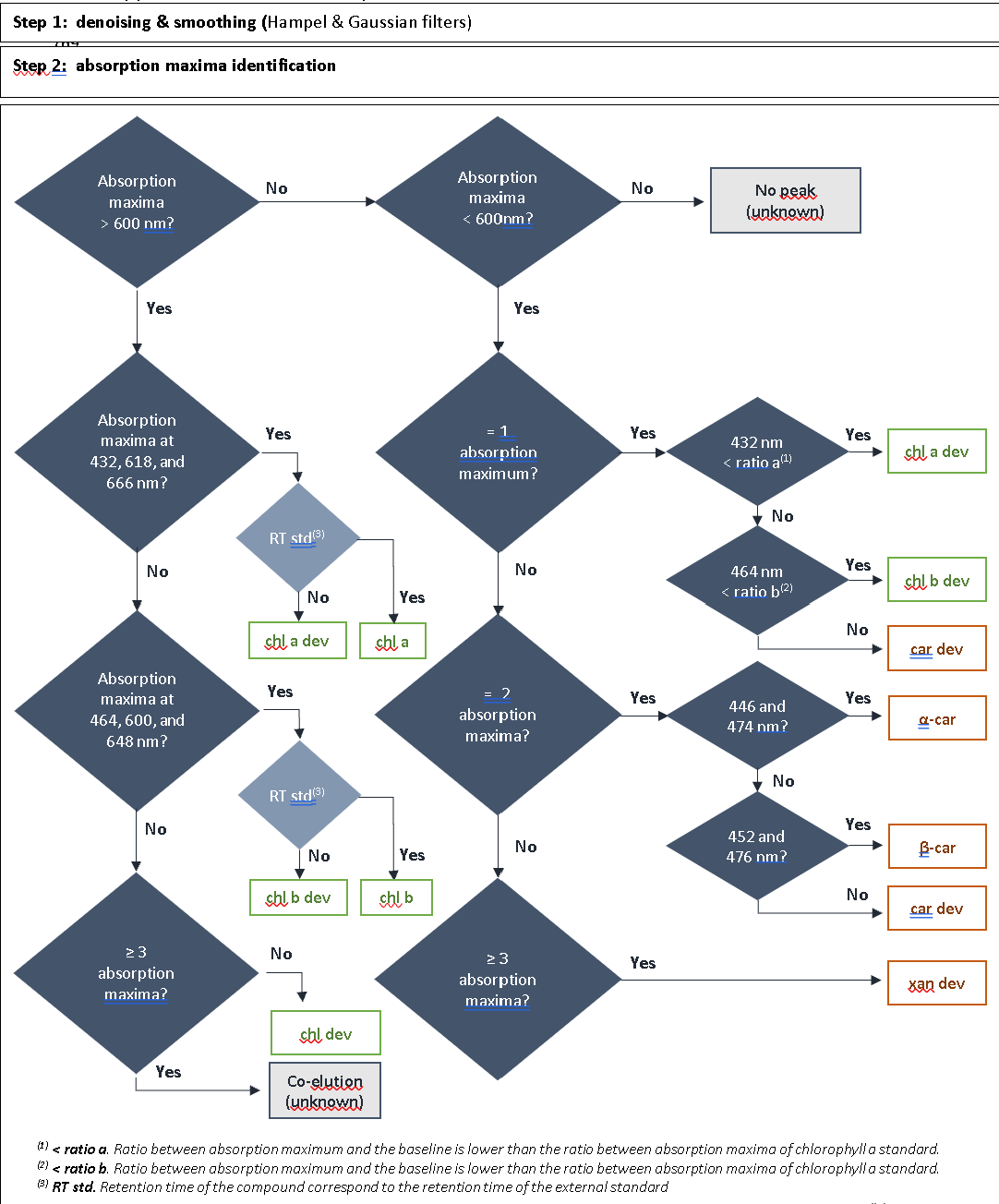
**

**Supplementary Figure 3 Workflow diagram of pigment compound identification and classification based on spectral absorption features and retention time.** Compounds are classified into three groups, chlorophylls *(green),* carotenoids *(orange)*, and unknown compounds *(grey)*. The chlorophyll group is subdivided into chlorophyll a (*chl a*) and chlorophyll a derivatives (*chl a dev*), chlorophyll b (*chl b*) and chlorophyll b derivatives (*chl b dev*) and other chlorophyll derivatives (*chl dev*). The carotenoid group is subdivided into α-carotene (*α-car*) β-carotene (β*-car*), carotene derivatives (*car dev*) and xanthophyll derivatives (x*an dev*). Spectra with either a low signal-to-noise ratio preventing compound class identification or mixed spectral features (co-elution) fall into the group of unknown compounds.


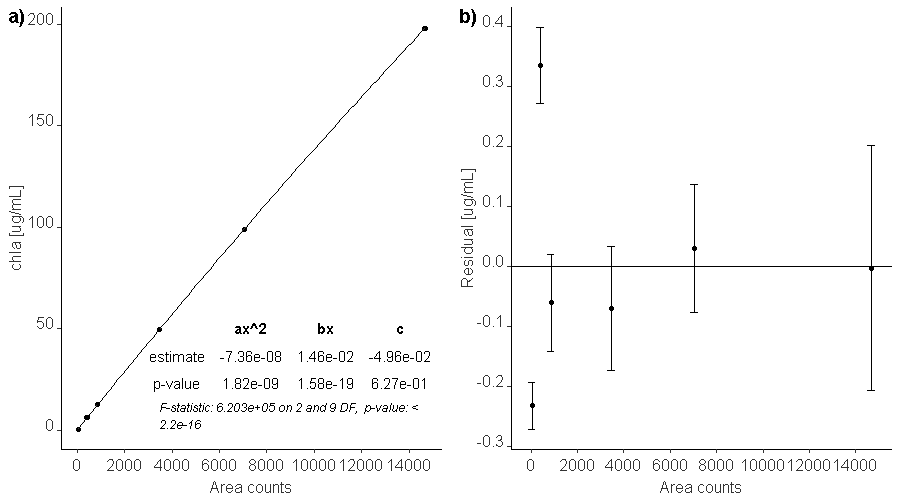


**Supplementary Figure 4. (a) Calibration curve and (b) residuals for *chl a*.**

To establish the calibration curve, 6 concentrations – 0.3, 6.2, 12.4, 49.5, 99, 198 µg/mL – of an analytical standard of *chl a* (Sigma Aldrich, purity >85%) were measured twice over two days *(1r x 2d)*. A quadratic response function, showing a significantly better fit than a linear regression *(ANOVA lack-of-fit test, F_9_=525.5, p=1.82e-09)*, was chosen for the calibration. The residuals calculated as the difference between observation and modelled values are randomly distributed, though the prediction of the model is weaker as the peak area falls below 500. Error bars correspond to the standard deviation (n=2).

Quadratic response function: [*chl* a] (μg.mL) = -7.360e^-8^ x^2^+ 1.457e^-2^ x + -4.961e^-2^


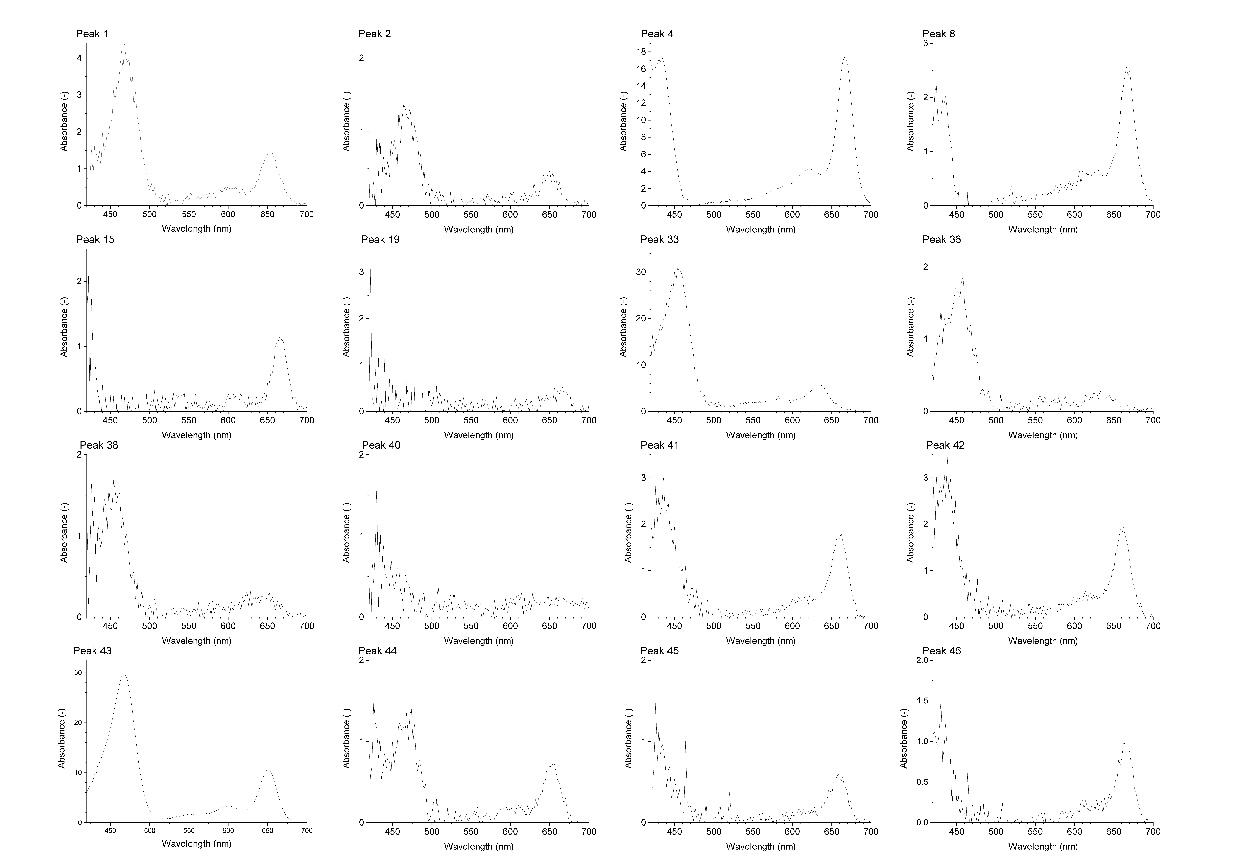

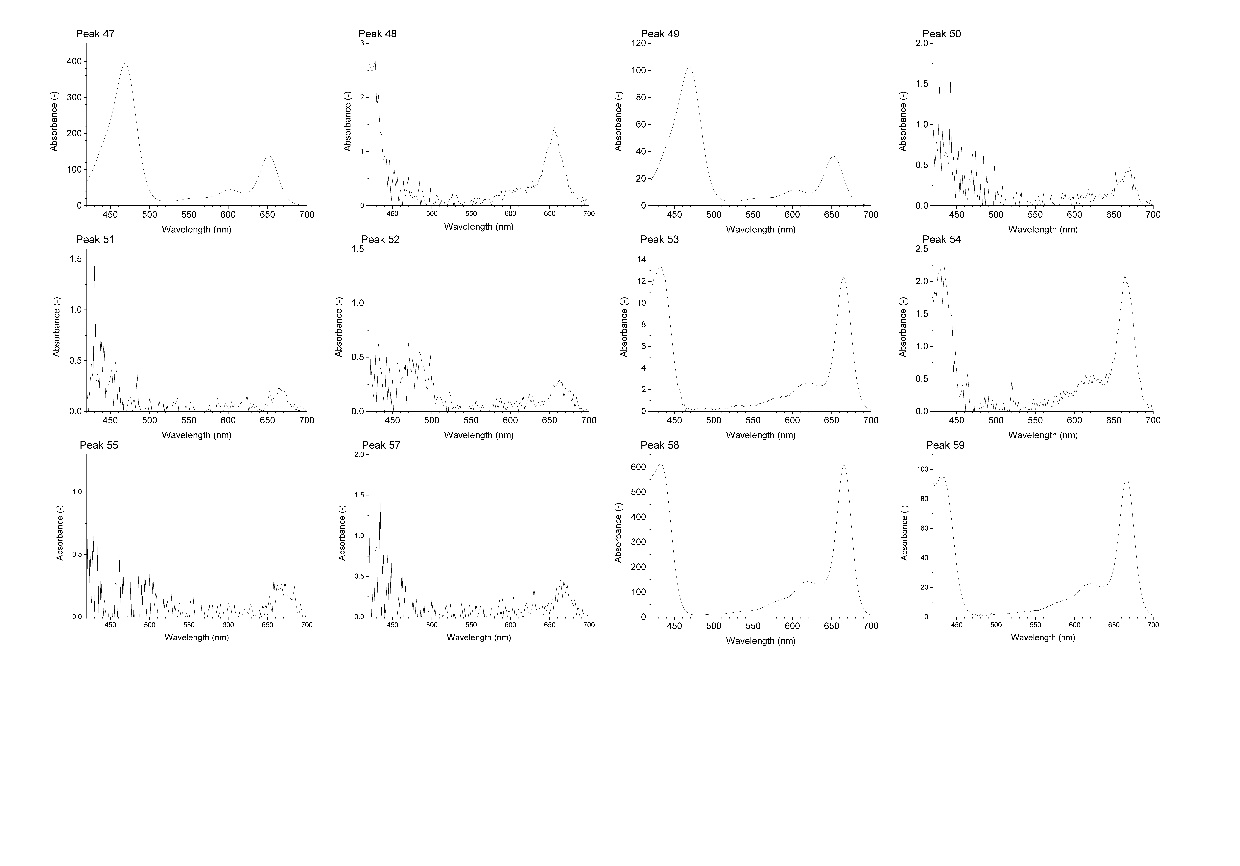


**Supplementary Figure 6.** Spectra of chlorophyll pigments.


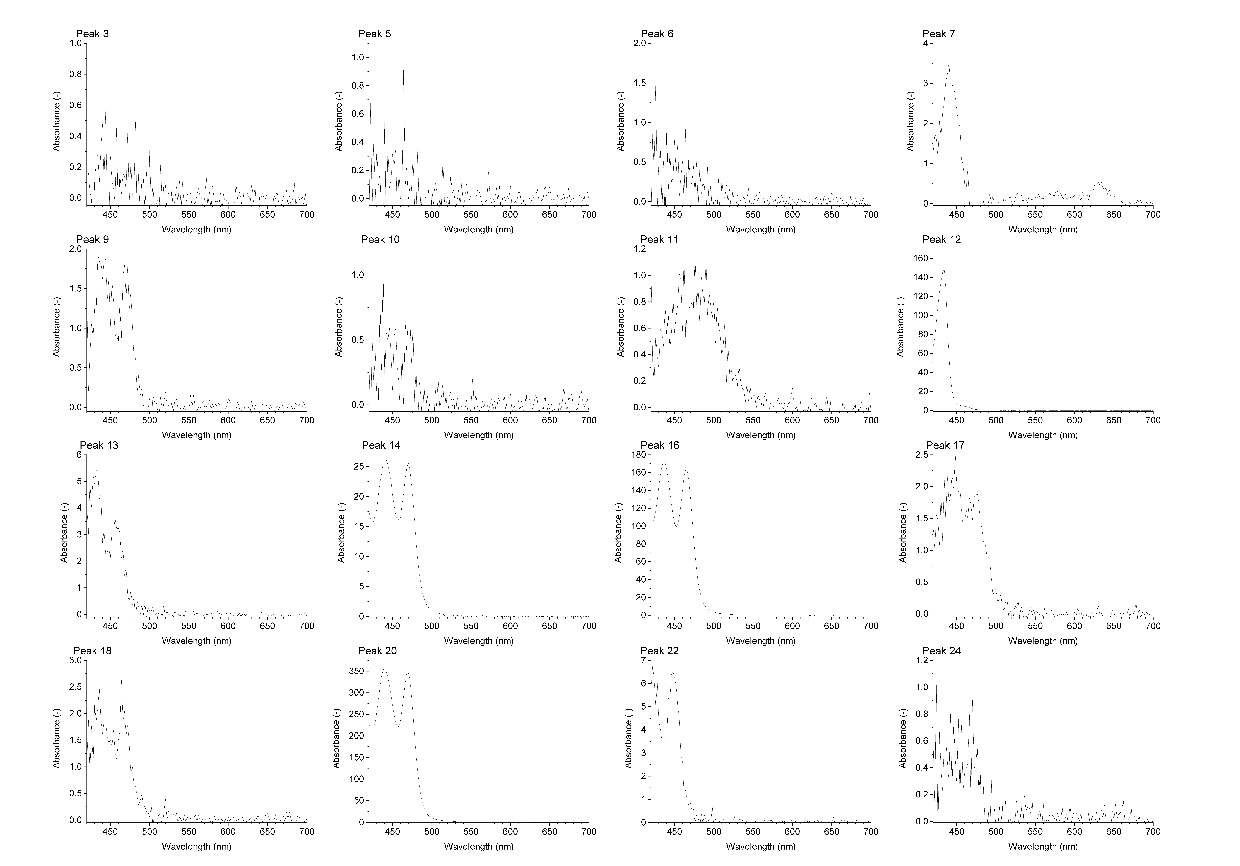

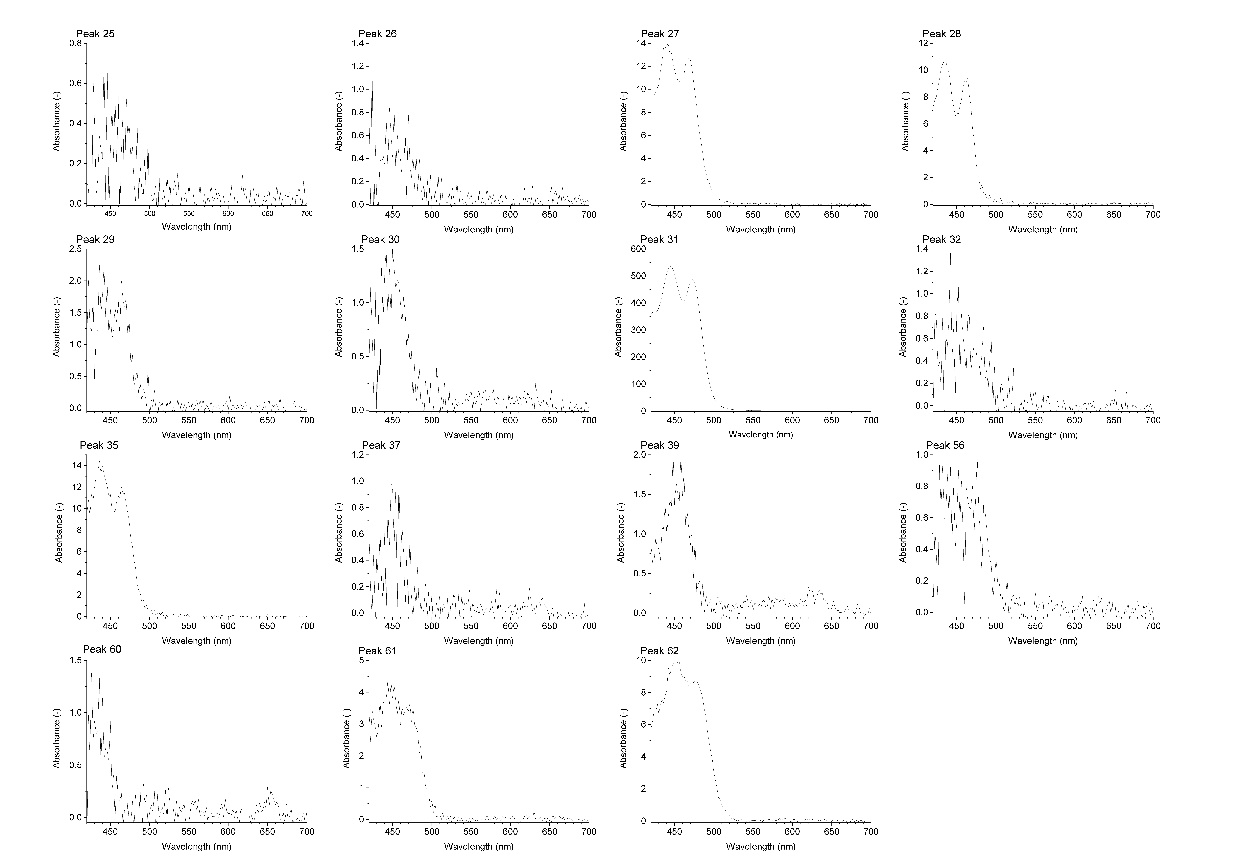


**Supplementary Figure 7.** Spectra of carotenoid pigments.

**Supplementary Figure 5.** HPLC chromatograms of four species (a) *Spinacia Oleracea*, (b) *Fagus sylvatica L.*, (c) *Carpinus betulus L.*, (d) *Tilia cordata Mill.* at 450 nm *(black)* and 665 nm *(red).*


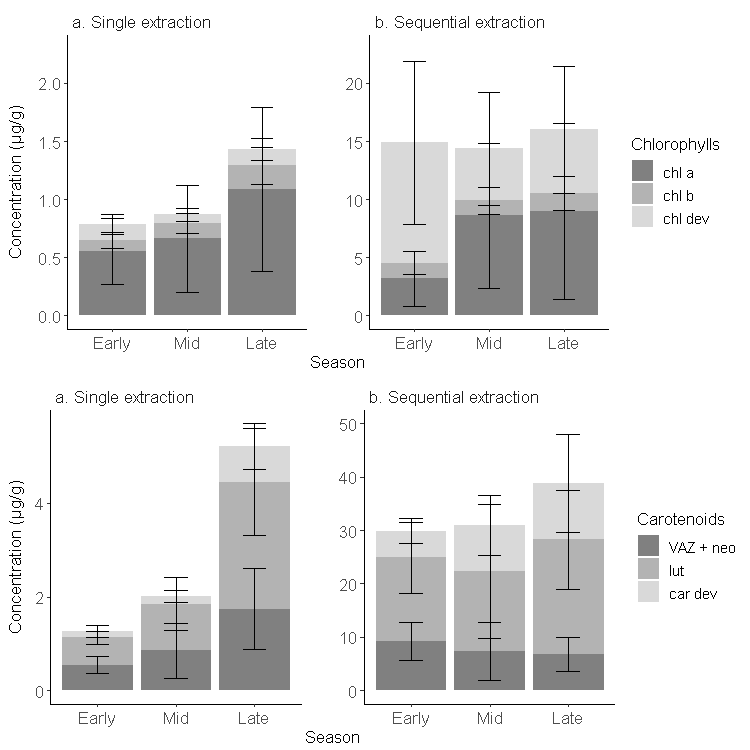


**Supplementary Figure 8.** Chlorophyll (a-b) and carotenoid (c-d) concentrations of beech leaves in early (May to June), mid- (July to August), and late (September to November) growing season 2018 obtained after a single extraction (a, c) and a sequential extraction (b, d). Error bars correspond to standard deviations (n=4).

**Supplementary Table 1. Chlorophyll concentrations in beech leaves reported in the literature between 1996 and 2021.** For each publication, the original chlorophyll concentration units as well as the extraction and characterization methods are reported. Extraction solvents included ethanol, (pure and aqueous) acetone), dimethyl sulfoxide (DMSO), and dimethylformamide (DMF). Characterization methods involved either a spectrophotometer or a high-performance liquid chromatography (HPLC), or a Soil Plant Analysis Development (SPAD) chlorophyll meter. ‘-‘ indicates that no concentration value was reported in the corresponding unit.

| **Ref.** | **Year** | **Extraction** | **Characterization** | **Concentrations *chl a+b*** | | | | |
| --- | --- | --- | --- | --- | --- | --- | --- | --- |
|  |  |  |  | μmol.m^-2^ | mg.m^-2^ | μmol.g^-2^dw | mg.g^-2^ dw | mg.g^-2^ fw |
| (Minotta and Pinzauti, 1996) | 1996 | single step - DMF | Spectrophotometer | - | - | - | 3.33-22.23 | - |
| (Polle *et al.*, 1997) | 1997 | single step - Acetone 80% | Spectrophotometer | - | - | 0.95-4.53 | - | - |
| (Tognetti *et al.*, 1998) | 1998 | single step - DMF | Spectrophometer | - | - | - | 4.5-12 | - |
| (García-Plazaola and J. M. Becerril, 2000) | 2000 | single step - Acetone 100% | HPLC | 200-400 | 180-360 | - | - | - |
| (García-Plazaola and José M. Becerril, 2000) | 2000 | single step - Acetone 100% | HPLC | 200-600 | 180-540 | - | - | - |
| (Lütz *et al.*, 2001) | 2000 | single step - Acetone 80% | HPLC | - | - | - | - | 0.7-2.5 |
| (Martínez-Ferri *et al.*, 2000) | 2000 | single step - Acetone | HPLC | - | - | - | - | - |
| (García-Plazaola and Becerril, 2001) | 2001 | single step - Acetone 100% | HPLC | - | - | - | - | - |
| (Lütz *et al.*, 2001) | 2001 | single step - Acetone 80% | HPLC | - | - | - | - | 0.7-2.5 |
| (Wittmann, Aschan and Pfanz, 2001) | 2001 | single step - DMSO | Spectrophotometer | 401-479 | 361-431 | - | - | - |
| (Wonisch *et al.*, 2001) | 2001 | multi step - Acetone 100% | HPLC | - | - | - | 2-7 | - |
| (Hansen, Fiedler and Rank, 2002) | 2002 | single step - Acetone 85% | HPLC | 300-600 | 270-540 | - | - | - |
|  | 2002 | single step- Acetone 80% | Spectrophotometer | - | - | - | 5.60-6.81 | - |
| (Peltzer, Dreyer and Polle, 2002) | 2002 | single step - Acetone 80% | Spectrophotometer | - | - | - | - | 1.38-2.12 |
| (Valladares *et al.*, 2002) | 2002 | Single step - DMSO (65°C) | Spectrophotometer | - | 100-400 | - | - | - |
| (Šprtová *et al.*, 2003) | 2003 | single step - Acetone 80% | Spectrophotometer & HPLC | 180-185 | 162-167 | - | - | - |
| (Wieser *et al.*, 2003) | 2003 | signle step - Acetone 100% | HPLC | 150-500 | - | 3-17 | - | - |
| (Fleischmann *et al.*, 2004) | 2004 | single step - Acetone 80% | HPLC | - | 0-3500 (chla) | - | 0.827-3.052 | - |
| (Láposi *et al.*, 2005) | 2005 | single step- Acetone 80% | Spectrophotometer | - | - | - | 1.44-2.98 | - |
| (Haberer *et al.*, 2007) | 2007 | multi step - Acetone 100% | HPLC | - | - | 3-14 | - | - |
| (Haisel *et al.*, 2006) | 2006 | single step - Acetone 100% | HPLC | 44-155 | 40-140 | - | - | - |
| (Gallé and Feller, 2007) | 2007 | single step - Ethanol:Water | Spectrophotometer | 100-250 | 90-225 | - | - | - |
| (Gielen *et al.*, 2007) | 2007 | single step - Acetone 80% | Spectrophotometer & HPLC | - | - | - | - | - |
| (Herbinger *et al.*, 2007) | 2007 | single step - Acetone 100% | HPLC | - | - | 2-15 | - | - |
| (Lichtenthaler *et al.*, 2007) | 2007 | single step - Acetone 80% | Spectrophotometer | 389-500 | 350-450 | - | 5-13 | - |
| (Sarijeva, Knapp and Lichtenthaler, 2007) | 2007 | single step - Acetone 100% | Spectrophotometer | - | 403-532 | - | 6.44-16.9 | 3.10-6.98 |
| (Peltzer and Polle, 2001) | 2008 | single step- Acetone 80% | Spectrophotometer | - | - | - | - | 2.18-3.46 |
| (Košvancová-Zitová *et al.*, 2009) | 2009 | none | SPAD | - | 200-240 | - | - | - |
| (Láposi *et al.*, 2009) | 2009 | single step- Acetone 80% | Spectrophotometer | - | - | - | 2.58-3.21 | - |
| (Panigada *et al.*, 2009) | 2009 | signle step - DMF | Spectrophotometer | - | 100-600 | - | - | - |
| (Closa, Irigoyen and Goicoechea, 2010) | 2010 | single step - Ethanol 96% (80°C) | Spectrophotometer | 167-422 | 150-380 | - | - | - |
| (Urban *et al.*, 2014) | 2014 | single step- Acetone 80% | Spectrophotometer | - | 290-350 | - | - | - |
| (Kraj, 2015) | 2015 | single step- Acetone 80% | Spectrophotometer | - | - | - | 0.4-6.60 | - |
| (Scartazza *et al.*, 2016) | 2016 | single step - Acetone 100% | HPLC | 420-426 | 378-384 | 5.57 - 16.73 | - | - |
| (Zajec *et al.*, 2016) | 2016 | single step - MeOH:Water:HCl | Spectrophotometer | - | 495-709 | - | - | - |
| (Kováč *et al.*, 2018) | 2018 | single step- Acetone 80% | Spectrophotometer | - | - | - | - | 1.4-2.1 |
